# Development of an Ethnic Identity Measure for Americans of Middle Eastern and North African Descent: Initial Psychometric Properties, Sociodemographic, and Health Correlates

**DOI:** 10.1007/s40615-020-00863-y

**Published:** 2020-09-24

**Authors:** Ken Resnicow, Minal R. Patel, Molly Green, Alyssa Smith, Elizabeth Bacon, Stefanie Goodell, Madiha Tariq, Asraa Alhawli, Nadia Syed, M. Lee Van Horn, Matthew Stiffler

**Affiliations:** 1 Department of Health Behavior & Health Education, University of Michigan School of Public Health, 109 Observatory Street, Room 3867 SPH I, Ann Arbor, MI 48109-2029 USA; 2 University of Michigan Rogel Cancer Center, Ann Arbor, MI USA; 3 ACCESS, 6450 Maple St., Dearborn, MI 48126 USA; 4 Department of Psychology, University of New Mexico, Albuquerque, NM USA

**Keywords:** Arab American, Middle Eastern, Ethnic identity

## Abstract

**Background:**

Southeast Michigan is home to the second largest Middle Eastern and North African (MENA) US population. There is increasing interest in understanding correlates of psychosocial outcomes and health behaviors in this growing population. One potentially important health correlate is ethnic identity (EI). This paper reports the development, validity, and initial correlates of a new measure of MENA identity named the MENA-IM.

**Methods:**

We used convenience sampling at locations frequented by individuals of MENA descent in southeast Michigan. We also measured EI centrality, religiosity, cultural mistrust, substance use, and health status to assess convergent and divergent validity. Exloratory and Confirmatory Factor Analysis identified three subscales, which were valid for both Arab and Chaldean respondents and were named (1) MENA cultural affiliation, (2) MENA media use, and (3) multicultural affiliation. We also created and tested a 20-item, single-factor version.

**Results:**

We obtained data from 378 adults, 73% of whom identified as Arab and 27% as Chaldean. MENA-IM scores were higher among older, lower-educated, lower-income, non-US born, and Arabic-speaking respondents. Arab respondents reported significantly higher scores than Chaldeans. MENA-IM scores were positively associated with EI centrality and religiosity. Higher MENA-IM scores were found among those not reporting use of marijuana, alcohol, and opiates. Higher MENA-IM scores were also found among those without a self-reported history of heart disease and among those with better mental health status.

**Discussion:**

The MENA-IM has strong psychometric properties and demonstrated initial evidence of convergent and discriminant validity. In general, values on the measure were associated with better psychosocial and health status. How the measure performs with MENA populations outside of Michigan and how it may relate to other health outcomes merit investigation.

## Introduction

The US population of individuals of Middle Eastern and North African (MENA) descent numbers between two and 3.7 million [1–3]. The lower population figure represents the US census estimate [1], while the upper estimate is adjusted upward for several undercounting factors, including the fact that individuals of MENA descent are considered White under current census criteria and MENA descent must be indicated under a separate ancestry question [2, 3]. By all accounts, the US MENA population has increased over the past 20 years [2].

The focus of this research is the MENA communities in southeast Michigan, who come from primarily Arabic-speaking countries. Southeast Michigan is home to the second largest MENA population in the USA (around 211,000 of Arab descent plus around 40,000 of Chaldean/Assyrian descent according to 2017 census estimates) with California having the largest population (around 307,000 of Arab descent plus around 31,000 of Chaldean/Assyrian descent by 2017 census estimates) [1]. Nationally, the MENA population is diverse, representing all 22 Arab League nations, with the primary countries of origin being Lebanon, Egypt, Syria, Iraq, Yemen, and Palestine as well as non-Arabic speaking countries such as Iran and Turkey. The Michigan MENA population is similar to the national population, although it has a higher percentage of Iraqis, many of whom are Christians of Chaldo-Assyrian descent and may not identify as Arab, as well as a smaller proportion of Iranians [1, 2, 4].

There has been increasing interest in understanding the prevalence and correlates of health and health behaviors among the MENA population [5–12]. Ethnic identity (EI) [13–15] and acculturation [16–19] [20] are two potentially important predictors of health status and health behavior, widely examined in other ethnic/racial subgroups, that have recently begun to be studied in MENA populations. Although these constructs encompass overlapping dimensions (e.g., they both incorporate affiliation with one’s cultural heritage and expression of cultural habits), they are not synonymous. EI can be defined as psychological, social, and behavioral affiliation with one’s familial ethnicity and cultural heritage. EI then can develop and manifest both in one’s native country as well as in one’s adopted country. EI, when discussed within the context of immigration and assimilation, is also referred to as “enculturation” [21, 22]. However, EI and enculturation can be further differentiated with the former including not only how much one retains their original beliefs, values, and habits, but also their desire to transmit their culture to future generations. Another important distinguishing element of EI is its relative centrality to overall identity, that is, how important these values, beliefs, and practices are to one’s broader definition of self. Acculturation, on the other hand, relates more to how much an individual has adopted the values and practices of their new host culture/country and not necessarily their desire to transmit their culture to future generations [20, 23, 24]. Whereas acculturation has generally been associated with increased health risk, due to both the stress of acculturation and the fact that immigrants often adopt less health promoting behaviors, e.g., diet and substance use, endemic in their new country [7, 20, 23], stronger ethnic identity on the other hand [24], has largely been shown to be a protective attribute [20]. Acculturation and ethnic identity can be conceived as orthogonal rather than inverse constructs, in that an individual could both acculturate to their new country and also retain a strong connection to their original culture/ethnicity.

There has been some work around measurement of acculturation/enculturation among Arab Americans, albeit with mixed findings. Jadalla and Lee adapted a measure, originally used with Hispanics, which they named the Acculturation Rating Scale for Arabic Americans-II, available both in Arabic and English (ARSAA-IIA, ARSAA-IIE) [25]. The measure yields two scores: attraction to American culture (i.e., acculturation) and attraction to Arab culture (i.e., enculturation). In one study using the ARSAA conducted among 267 Arab American women in California [26], greater attraction to American culture (acculturation) was associated with more positive health behaviors, whereas attraction to Arabic culture was unrelated to health behaviors. A second study among 297 Arab Americans surveyed in California using the ARSAA measure found that both attraction to American culture and Arab culture were associated with positive health behaviors. Specifically they found that attraction to American culture was associated with higher levels of physical activity, whereas attraction to Arabic culture was associated with better stress management and diet [27]. Finally, in a separate report of the same 297 Arab Americans, [12] attraction to American culture was associated with higher alcohol use, whereas attraction to Arab culture was associated with higher cigarette smoking.

The impact of acculturation on health behaviors may manifest differently for Arab Americans than other immigrant groups. In a sample of 1400 Arab Americans surveyed in California [9], of the 10 health behaviors and health conditions examined, second- and third-generation AAs differed little from first-generation immigrants. The authors conclude there was “little evidence for the immigrant health paradox among immigrants from Arabic-speaking countries…”. It is possible that ethnic identity may be a more powerful predictor of health status than immigrant generation among Arab Americans, or it may buffer the deleterious effects of acculturation.

Together, these prior studies indicate a need to examine the role of EI among MENA individuals. This manuscript reports the development, psychometric properties, and initial sociodemographic and health correlates of a new measure of MENA ethnic identity we call the MENA-IM.

## Methods

All study procedures were approved by the University of Michigan human subjects research committee in accordance with the Helsinki Declaration of 1975, as revised in 2000. Informed consent was obtained from all participants included in the study.

### Scale Development

The MENA-IM was adapted from prior ethnic identity measures developed for African Americans [14, 28, 29] and Latinos [18, 19] (see Table 1). The key domains of EI we aimed to assess were (1) connection to prior country/region of origin (sample item: *I keep up with political activities in the Middle East and North Africa*), (2) desire to preserve and transmit family culture and traditions (sample item: *It is important for Arab American people to educate their children about Arab/Arab American art, history, music, and literature*), (3) centrality of Arab American and Chaldean identity (sample item: *Both in my public and private thoughts, being Arab American is an important part of who I am*), (4) respect for other cultures or multiculturalism (sample item: *I care deeply about the needs of other groups such as Native Americans,* etc.), and (5) Arab American media use (sample item: *When I watch television, I usually watch Arabic television shows, such as ART and MBC*). Modifications from the African American and Latino versions were required to make the measure applicable for Arab Americans in general, and in some instances to tailor survey items for Michigan. For example, media consumption included popular international Arabic television networks such as ART and MBC but also local Michigan radio stations (e.g., CINA) and regional newspapers (e.g., Arab American News). All items were answered along a four-point continuum ranging from strongly disagree to strongly agree. Higher scores indicate stronger ethnic identity.

**Table 1:** Rotated^^^ factor loadings for the 20-item MENA Identity Measure (MENA-IM) for Arab and Chaldean

Item	Arab respondents			Chaldean respondents		
	Total 20-item scale alpha = .922			Total 20-item scale alpha = .903		
	Factor 1: cultural affiliation	Factor 2: media use	Factor 3: multicultural	Factor 1: cultural affiliation	Factor 2: media use	Factor 3: multicultural
Internal consistency alpha	.81	.88	.83	.86	.88	.80
1. Many things that are important to me are connected to my Arab American identity.	.86			.82		
2. Both in my public and private thoughts, being Arab American is an important part of who I am.	.80			.84		
3. Many things that make me happy are connected to the fact that I am Arab American.	.80			.74		
4. In my private thoughts, I think of myself more as Arab American than American.	.79			.85		
5. Most of my friends are Arab American.	.64			.64		
6. When I watch television, I usually watch Arabic television shows, such as ART and MBC.		.86			.89	
7. When I listen to the radio, I usually listen to Arab American radio shows, e.g., CINA.		.83			.84	
8. When I look for news, I read mostly Arabic news such as The Arab American News and the Dearborn Facebook page.		.81			.90	
9. I care deeply about the needs of other groups such as Native Americans, African Americans, Latinos, and Asian Americans.			.88			.91
10. I feel strongly about international human rights issues in places such as Africa.			.83			.68
11. I respect the cultural traditions of many groups, e.g., Native Americans, African Americans, Latinos, and Asian Americans.			.72			.84
12. It is important to be involved in the Arab American community.	Ex	Ex	Ex	Ex	Ex	Ex
13. It is important for Arab American people to educate their children about Arab/Arab American art, history, music, and literature.	Ex	Ex	Ex	Ex	Ex	Ex
14. A thorough knowledge of Arab and Arab American history is very important for our community today.	Ex	Ex	Ex	Ex	Ex	Ex
15. I have a strong sense of belonging to the Arab American community.	Ex	Ex	Ex	Ex	Ex	Ex
16. I feel a strong emotional connection to the Middle East or North Africa.	Ex	Ex	Ex	Ex	Ex	Ex
17. Arab Americans should give their children Arabic names.	Ex	Ex	Ex	Ex	Ex	Ex
18. I keep up with political activities in the Middle East and North Africa.	Ex	Ex	Ex	Ex	Ex	Ex
19. It is important for Arab Americans to get back to their Middle Eastern/North African roots.	Ex	Ex	Ex	Ex	Ex	Ex
20. It is important for us to eat Middle Eastern/North African food at home.	Ex	Ex	Ex	Ex	Ex	Ex

*Ex* excluded from the common three-factor solution, but included in the full 20-item version

^^^Varimax rotation

The measure was translated into Arabic using an iterative process, as per recommended methods [30]. We began by having a bilingual Arabic language expert translate the English version into Modern Standard Arabic. Translations were reviewed by bilingual professionals from our community partners. After each review, appropriate modifications were made.

After the professional reviews, we conducted cognitive pretesting of the MENA-IM and other new measures among five primarily Arabic-speaking individuals: two men and three women, ages 45–60. The interviews were conducted in both Arabic and English and were facilitated by study team members, one of whom was fluent in both languages.

Participants of the cognitive interviews suggested several modifications to the ethnic identity items. Specifically, they felt that several items could better distinguish between internal manifestations of identity (thoughts and feelings) and external behavioral items (outward expressions). To address this recommendation, we added the phrase “in my private thoughts” to relevant items. They also recommended adding “North Africa” or “North African” in addition to Arab American and Middle Eastern.

After the cognitive interviewing, the full electronic version was tested among three participants (2 in English and 1 in Arabic) and the paper version was tested among two participants (1 English, 1 Arabic). Further minor revisions were made based on the second round of pretesting feedback.

#### Other Measures

To explore concurrent validity, divergent validity, and predictive validity of the MENA-IM scale, we compared scores with other identity-related variables as well as health behaviors and health status.

*Racial mistrust* was measured using the mean of two items adapted from a prior measure [28]. The two items, each answered along a 1 (strongly disagree) to 4 (strongly agree) continuum were (1) *When I think about culture/race relations in America, I get upset* and (2) *The United States government is trying to make things better for Arab Americans*. The later item was reverse-coded prior to computing the scale score so that higher values for the two-item mean indicated greater mistrust.

*Centrality of MENA identity* was assessed with a single item, adapted from our prior work [28]. The item queried, “how important is being Middle Eastern/North African to your overall identity?” Responses ranged from with zero (not at all important) to 10 being (very important). This item is intended to serve as a brief measure of global ethnic identity and was assumed to be positively correlated with MENA-IM scores.

*Mental health symptoms* were assessed with the four-item PHQ-4 [31], which asked, “how often have you been bothered by any of the following problems?” (1) Little interest or pleasure in doing things; (2) feeling down, depressed, or hopeless; (3) feeling nervous, anxious, or on edge; and (4) not being able to stop or control worrying, all of which were answered with a 1–4 scale, with 4 being “not at all” and 1 being “nearly every day.” Higher scores are indicative of better mental health status. Alpha for the four items in our sample was 0.91.

*Religiosity* was measured with a three-item scale adapted from Krause [32]**;** 1) God/Allah put me in this life for a purpose, 2) God/Allah has a specific plan for my life, and 3) God/Allah has a reason for everything that happens to me. Responses were 1 “strongly disagree” through 4 “strongly agree”. Alpha for the three items in our sample was 0.96.

Health behaviors assessed included *current cigarette use*, defined as consuming at least 100 cigarettes lifetime and currently smoking on at least some days in the past month [33]; *past month alcohol use* defined as consuming at least one drink of any alcoholic beverage at least once in the past 30 days [33]; *past month marijuana*, defined as any use in the past 30 days; *past year Hookah use*, defined as use on greater than 2 occasions in the past year. [8, 34] *Lifetime pain medication use* was queried with an item from the 2017 Youth Risk Behavior Survey [35]; “During your life, how many times have you taken prescription pain medicine without a doctor’s prescription or differently than how a doctor told you to use it?” (count drugs such as codeine, Vicodin, OxyContin, hydrocodone, and Percocet). Use was considered more than 2 times in one’s lifetime.

Self-reported *medical history* was assessed by asking if the respondent had ever been diagnosed with (1) cancer, (2) diabetes or high blood sugar, (3) high blood pressure or hypertension, or (4) heart condition such as heart attack, angina, or congestive heart failure. Each variable was answered NO (0) or YES (1).

*Demographic variables* assessed included age (collapsed into 4 groups: 18–35, 30–45, 45–65, and > 65), household income (collapsed into 4 groups: under $10,000; $10,000 to $49,999; $50,000 to $99,999; and > $100,000), education (collapsed into 4 groups: high school or less, some college, college graduate, graduate school or higher), religion (Muslim or Christian), native country (US-born and not US-born), Arabic spoken at home (yes or no), and Arab or Chaldean identity.

### Survey Administration

Surveys were distributed at 12 settings, across three Michigan counties, that included two supermarkets frequented by the MENA community, one health clinic serving as a predominantly MENA population, one health clinic serving a predominantly Chaldean population, a state university with a high number of Arab American students, four mosques with a high proportion of Yemeni and Lebanese worshippers, two Chaldean churches, and a recreation center frequented by Lebanese youth.

Participants were given the option of completing surveys using pen and paper or online forms (tablet provided), with or without assistance, in English or Arabic. For those opting to complete surveys at home, we provided a self-addressed stamped envelope or a web address to complete the online version. Both paper and electronic surveys required active consent and testament that the respondent was over 18 and self-identified as Arab or Chaldean. Data collectors, many of which were fluent in both English and Arabic, were trained in interviewing by study staff. Participants received a $25.00 gift card after completing their survey.

### Scale Construction and Psychometric Properties

The 20 MENA-IM items were examined initially through exploratory factor analysis using principle components extraction, with Varimax rotation in SPSS 25 [36]. One of our goals was to create a scale that could be used for both Arab and Chaldean populations, i.e., we wanted to create a factor structure that was invariant between Arab and Chaldean respondents. We therefore identified an initial three-factor solution that appeared conceptually and statistically to fit for both the Arab and Chaldean respondents. This solution utilized 11 of the original 20 items. This three-factor solution was tested for invariance between Arab and Chaldean respondents through confirmatory factor analysis (CFA; conducted in Mplus 7).

For the CFA, items were all treated as ordinal, and measurement invariance was compared between a configural invariance model (Chi-square = 277.18, df = 82, RMSEA = .09, TLI = .98, CFI = .98) and a model which simultaneously constrained factor loadings, item thresholds, and scaling parameters (Chi-square = 218.41, df = 120, RMSEA = .08, TLI = .98, CFI = .98). While the constrained model fit was somewhat weaker than the configural invariance model (Chi-square = 82.47, df = 38), the factor structure parameters were essential similar for both groups. Given the empirical fit data, combined with the high interpretability of the three-factor solution, we proceeded with the common three factors for both the Arab and Chaldean respondents. Additional results of the exploratory and confirmatory factor analyses are available from the first author upon request.

The three factors were named MENA CULTURAL AFFILIATION (5 items), MENA MEDIA USE (3 items), and MULTICULTURAL AFFILIATION (3 items). Individual items and their factor loadings can be found in Table 1. Correlations between the three subscales ranged from .13 (between Factors 2 and 3), .36 (between Factors 1 and 2) to .58 (between Factors 1 and 3).

In addition to presenting results for the common three-factor solution, we also present results for the full 20-item scale, as some practitioners and researchers may not be working with both Arab and Chaldean populations in the same study and because the broader 20-item measure may be preferable under some situations. Scale scores for the three common factors and the overall 20-item scale measure were computed by taking the mean of items in that scale.

### Statistical Analyses

We hypothesized that MENA-IM scores would be positively associated with the EI centrality item as well as the religiosity and mental health scales. Because we conceptualize ethnic identity as only partially overlapping with these constructs, we hypothesized that the associations, while significant and positive, would be of modest magnitude, thereby indicating both convergent and discriminant validity. We did not have a priori directional or magnitude hypotheses regarding the relationship between MENA-IM with cultural mistrust. We also hypothesized that higher MENA-IM scores would be associated with lower rates of substance and self-reported disease.

We first present correlations between continuous variables (e.g., other identity measures and mental health symptoms) with MENA-IM scores (Table 3) and then multiple regression results adjusting for income, education, age, gender, and place born (Table 4). For categorical independent variables, we present mean MENA-IM scores by sociodemographic variables, without covariate adjustment (Table 5). For health behaviors and medical history, which were all dichotomous variables, we report multivariate regression, using MENA-IM scores as the primary dependent variable and substance use and health history as the independent variables, adjusting for income, education, age, gender, religiosity, and place born (Table 6).

## Results

We obtained responses from 394 individuals, 79% of whom completed their survey in English (see Table 2). The sample comprised 73% who identified as being of Arab descent and 27% who identified as Chaldean. The sample was majority female (63%). About half were between ages 18 and 25 (48.8%). Most had less than a college education (58%) and most reported household income below $50,000 (69%). Two thirds were Muslim, and 33% were Christian. Of the Christian respondents, most (89%) were Chaldean (data not shown). The most common paternal countries of origins were Lebanon (40.8%), Iraq (36.5%), and Yemen (14.8%).

**Table 2:** Sample demographics (*n* = 394)

	Percent
Gender	
Male (*n* = 146)	37.1
Female (*n* = 248)	62.9
Education	
High school or less (*n* = 129)	33.2
Some college (*n* = 95)	24.4
College graduate (*n* = 120)	30.8
Graduate school (*n* = 45)	11.6
Age	
18–35 (*n* = 188)	48.8
30–45(*n* = 90)	23.4
45–65(*n* = 86)	22.1
> 65(*n* = 22)	5.7
Income	
Under $10,000 (*n* = 59)	15.4
$10,000 to $49,999 (*n* = 204)	53.4
$50,000 to $99,999 (*n* = 76)	19.9
$> 100,000 (*n* = 43)	11.3
Born in the USA	
Yes (*n* = 129)	32.9
No (*n* = 263)	67.1
Origin country of father	
Lebanon (*n* = 160)	40.8
Iraq (*n* = 143)	36.5
Yemen (*n* = 58)	14.8
Egypt (*n* = 11)	2.8
Palestine (n = 9)	2.3
Syria (*n* = 6)	1.5
Other (n = 5)	1.4
Ethnic identity	
Arab (*n* = 273)	72.6
Chaldean (*n* = 103)	27.4
Religion	
Muslim (*n* = 252)	64.3
Christian (*n* = 130)	33.2
Other (*n* = 10)	2.5
Arabic spoken at home	
Yes (*n* = 340)	89.0
No (*n* = 42)	11.0

Alpha for the full 20-item scale was .92 and .90 for Arab and Chaldean respondents, respectively. Alpha for the cultural affiliation factor was .81 and .86 for Arab and Chaldean respondents. Alpha for the media use factor was .88 for both Arab and Chaldean respondents. And for the third factor, alpha was .83 and .80, for Arab and Chaldean respondents.

The means for the three factors and the 20-item total scale can be found on the bottom row of Table 5. The 20-item scale had an overall mean of 2.93, (median = 3.0) a standard deviation of 0.60, and an observed range of 1.0 to 4.0 (data not shown). The means for all three factor scores as well as the 20-item overall scale were all significantly different (*p* < .01) between Arab and Chaldean respondents, with Chaldeans scoring lower than Arab respondents (data not shown).

As shown in Table 3, for Arab respondents, all three factor scores were significantly and positively correlated with the single item EI centrality item, whereas among Chaldean respondents, this item was significantly correlated with Factors 1 and 3. The single centrality item was also significantly correlated with the 20-item total score. Religiosity was significantly correlated with Factor 1 for Arab respondents, with Factors 1 and 3 for Chaldeans, and with the 20-item total score. Mental health was significantly correlated with Factor 3 for Arab respondents as well as the 20-item total score. Finally, mistrust of government was positively and significantly correlated with Factors 1 and 3 for Arab respondents and with the 20-item total score. Mistrust was negatively correlated with the media use factor among both Arab and Chaldean respondents.

**Table 3:** Bivariate correlations: MENA-IM factor scores and total score with identity constructs for Arab and Chaldean respondents and total sample

	Arab			Chaldean			All
	Factor 1: cultural affiliation	Factor 2: media use	Factor 3: multicultural	Factor 1: cultural affiliation	Factor 2: media use	Factor 3: multicultural	20-item total scale
Arab American centrality	0.55^##^	.15^#^	.41^##^	.42^##^	.04	.22^#^	0.50^##^
Religiosity	0.12^#^	.02	.12	.55^##^	.05	.46^##^	0.24^##^
Mental health status*	0.06	.05	.13^#^	.10	.05	.17	0.13^#^
Mistrust	0.25^##^	− .21^#^	.26^##^	− .02	− .25^##^	− .13	0.15^#^

^#^*p* < .05

^##^*p* < .01

*Mean of 4 items; higher scores indicate more positive mental health status

All variables that had correlations at least ± 0.20 or greater in Table 3 were still significantly associated in regression analyses, which controlled for age, gender, income, education, and place of birth (see Table 4). Correlations that were significant, but were less than ± .20, were no longer significant in the multivariate model, suggesting that some of the associations detected in the correlation analyses were confounded by these covariates. Two exceptions were the association between Arab American centrality and Factor 3 for Chaldeans (which became nonsignificant in multivariate analyses) and religiosity and Factor 2 for Chaldeans (which became significantly inversely associated in multivariate analyses).

**Table 4:** Adjusted^^^ regression coefficients: MENA-IM factor scores and total scale score with identity constructs for Arab and Chaldean respondents and total sample

	Arab			Chaldean			All
	Factor 1: cultural affiliation	Factor 2: media use	Factor 3: multicultural	Factor 1: cultural affiliation	Factor 2: media use	Factor 3: multicultural	20-item total scale
Arab American centrality	.49^##^	− .07	.15#	.39^##^	.05	− .03	.53^##^
Religiosity	.07	− .01	.10	.44^##^	− .26^#^	.29^#^	.26^##^
Mental health status*	.02	.12	.03	.04	.19	.10	.16^##^
Mistrust	.20^##^	− .21^##^	.20^##^	.04	− .08	− .14	.22^##^

^^^Linear regression adjusted for age, income, education, gender, and foreign-born vs. born in the USA

^#^*p* < .05

^##^*p* < .01

*Mean of 4 items; higher scores indicate more positive mental health status

As shown in Table 5, neither factor scores nor the 20-item total score differed by gender. Factor 2 scores for both Arab and Chaldean respondents, Factor 3 scores for Chaldean respondents, and the 20-item total score were significantly, inversely associated with education, that is, higher education was associated with lower scores. Factor 2 for Arab respondents and the 20-item total score were positively associated with age, that is, older respondents showed higher mean scores. The association of Factor 3 with age for Chaldeans while significant is difficult to interpret as values were highest in the 3rd age band. For income, Factor 2 scores for both Arab and Chaldean respondents, decreased as income increased. The association of Factor 3 with income for Chaldeans while significant was again difficult to interpret as values were highest in the 3rd income band. Factor 2 scores for both groups and the total 20-item score were significantly higher among those born outside the USA. Paternal country of birth was significantly associated with Factor 1 for Arab respondents as well as the total 20-item scale score. Highest scores were observed for those of Lebanese and Syrian descent. For Chaldeans, the entire sample reported being of Iraqi paternal lineage. Muslims showed higher scores than Christians for Factors 1 and 3 among Arab respondents and for the 20-item total scale score. Again, comparisons by religion were possible for Chaldeans as the entire sample reported being Christian. Finally, scores were higher among those who reported speaking Arabic at home, except for Factors 2 and 3 for Chaldean respondents.

**Table 5:** Mean MENA-IM factor scores by demographic variables^^^

	Arab			Chaldean			All
	Factor 1: cultural affiliation	Factor 2: media use	20-item total scale	Factor 1: cultural affiliation	Factor 2: media use	Factor 3: multicultural	20-item total scale
Gender							
Male	3.2	2.3	3.2	2.6	2.1	3.0	2.9
Female	3.3	2.4	3.4	2.8	2.0	3.1	3.0
Education		^##^			^##^	^#^	^#^
High School or less	3.3	2.7	3.4	2.7	2.5	2.7	3.0
Some college	3.2	2.2	3.2	2.8	2.4	3.0	2.9
College graduate	3.2	2.2	3.3	2.8	1.5	3.2	2.9
Graduate school	3.0	1.9	3.3	2.9	1.6	3.4	2.8
Age		^##^				^#^	^##^
18–35	3.2	2.0	3.3	2.7	1.8	3.1	2.9
30–45	3.1	2.4	3.4	2.6	1.9	2.8	2.8
45–65	3.4	2.9	3.4	3.1	2.2	3.3	3.1
> 65	3.5	2.8	3.4	2.7	2.4	3.0	3.1
Income		^##^			^##^	^#^	
Under $10,000	3.2	2.5	3.4	2.6	2.5	2.8	3.0
$10,000 to $49,999	3.2	2.5	3.3	2.8	2.5	3.1	3.0
$50,000 to $99,999	3.2	2.1	3.3	2.9	1.3	3.5	2.8
$> 100,000	3.5	1.8	3.5	2.6	1.3	3.0	2.8
Born in the USA		^##^			^##^		^#^
Yes	3.2	2.0	3.3	2.9	1.2	3.2	2.8
No	3.2	2.5	3.4	2.8	2.2	3.0	3.0
Origin country of father^^^	^#^						^##^
Lebanon	3.4	2.4	3.4	–	–	–	3.1
Iraq	3.1	2.3	3.3	2.8	2.0	3.0	2.7
Yemen	3.1	2.4	3.2	–	–	–	3.0
Palestine	2.9	1.9	3.0	–	–	–	2.7
Egypt	3.1	2.8	3.0	–	–	–	3.0
Syria	3.7	1.9	3.6	–	–	–	3.2
Religion	^#^		^#^				^##^
Muslim	3.3	2.4	3.4	–	–	–	3.1
Christian	2.7	2.0	2.8	2.8	2.0	3.1	2.7
Arabic spoken at home	^#^	^#^	^#^	^#^			^##^
No	2.7	1.6	2.8	2.5	1.8	3.0	2.5
Yes	3.2	2.4	3.3	2.9	2.1	3.1	3.0
Total	3.2	2.3	3.3	2.8	2.0	3.1	2.9

^^^Only reported for cells with > 5 individuals

^#^Group means differ *p* < .05

^##^Group means differ *p* < .01

As shown in Table 6, in multivariate analyses adjusting for age, income, education, religiosity, and place of birth, Factor 1 and Factor 3 scores among Arab respondents and the 20-item total score were significantly higher among those reporting no past-month marijuana use. Factor 1 and Factor 2 scores among Chaldean respondents and the 20-item total score were significantly higher among those who reported no past month alcohol use. Factor 1 and Factor 3 scores among Chaldean respondents were significantly higher among those who reported no lifetime opiate use.

**Table 6:** Adjusted MENA-IM scores by substance use behaviors and self-report illness

	Arab			Chaldean			All
	Factor 1: cultural affiliation	Factor 2: media use	Factor 3: multicultural	Factor 1: cultural affiliation	Factor 2: media use	Factor 3: multicultural	20-item total scale
Hookah past year	NS	NS	NS	NS	NS	NS	NS
No	3.2	2.2	3.3	2.9	1.9	3.1	2.9
Yes	3.3	2.4	3.4	2.5	2.1	2.9	3.0
Cigarette past month	NS	NS	NS	NS	NS	NS	NS
No	3.2	2.3	3.3	2.8	2.0	3.1	2.9
Yes	3.2	2.3	3.4	2.6	2.0	2.8	2.8
Marijuana past month	^#^	NS	^#^	NS	NS	NS	^#^
No	3.3	2.3	3.4	2.8	2.0	3.1	2.9
Yes	2.7	2.1	2.8	2.7	1.8	3.3	2.5
Alcohol past month	NS	NS	NS	^#^	^#^	NS	^##^
No	3.3	2.3	3.4	2.9	2.1	3.1	3.0
Yes	2.9	2.0	3.3	2.6	1.7	3.0	2.6
Lifetime pain medication without prescription	NS	NS	NS	^#^	NS	^#^	NS
No	3.3	2.3	3.3	2.8	2.0	3.1	2.9
Yes	3.1	2.4	3.3	2.2	1.6	2.5	2.9
History of cancer	NS	NS	NS	NS	NS	NS	NS
No	3.2	2.3	3.3	2.8	1.9	3.0	2.9
Yes	3.5	2.4	3.0	3.1	2.3	3.6	3.1
History of heart disease	NS	NS	^#^	NS	NS	^#^	^#^
No	3.2	2.3	3.4	2.8	1.9	3.1	2.9
Yes	2.9	2.0	2.7	2.9	2.0	2.4	2.6
History of high blood pressure	NS	NS	NS	NS	NS	NS	NS
No	3.3	2.3	3.4	2.8	1.9	3.1	2.9
Yes	3.0	2.3	3.2	2.8	2.1	3.0	2.9
History of diabetes	NS	NS	NS	NS	NS	NS	NS
No	3.2	2.3	3.4	2.8	2.0	3.1	3.0
Yes	3.1	2.3	3.2	2.8	1.8	2.9	2.8

Adjusted for age, income, education, gender, religiosity, and foreign-born vs. born in the USA

*NS* no statistically significant difference between Yes and No groups

^#^Group means differ *p* < .05

^##^Group means differ *p* < .01

Those with a self-reported history of heart disease had higher scores on Factor 3 (both Arab and Chaldean) and the 20-item total scale than those reporting no history of heart disease. No other substance use behaviors or chronic diseases were associated with identity scores.

## Discussion

We report the initial psychometrics and validity of a new measure of ethnic identity developed for Americans of MENA descent. The 20 items yielded three subscales with similar factor structure for both Arab and Chaldean respondents as well as a 20-item, single-factor version. The three subscales assess discrete elements of ethnic identity: (1) positive cultural affiliation (five items), (2) MENA media use (3 items), and (3) multicultural attitudes (3 items). These scales showed high internal consistency.

Scale scores were significantly associated with identity centrality and religiosity, in the expected direction. The moderate magnitude of these associations, correlations ranging from .15 to .55, indicates that while related, these constructs tap different dimensions of identity. We consider this pattern of results evidence of both convergent and discriminant validity of the MENA-IM.

Mistrust, while positively associated with Factor 1 for Arab respondents as well as the 20-item total score for the full sample, was negatively correlated with the media use factor among both Arab and Chaldean respondents. That is, those who reported higher MENA media consumption had lower scores on the mistrust measure. It appears that consumption of MENA media lowers mistrust toward the US government and decreases perceptions of discrimination. It is possible that these media sources portray US politics in a more positive light or perhaps individuals who obtain their news and entertainment from these sources are less likely to be exposed to negative aspects of US culture. Why the cultural affiliation factor was positively associated with mistrust whereas the media use factor was negatively associated with mistrust merits elucidation.

With regard to demographic correlates, higher scores on several subscales as well as the overall 20-item scale were found among older, less-educated, lower-income, foreign-born, Arab American (compared with Chaldeans), Arabic-speaking, and Muslim compared with Christian respondents. MENA-IM scores were unrelated to gender.

The fact that older and non-US-born respondents had higher MENA-IM scores suggests that ethnic identity may weaken among subsequent generations of MENA immigrants. Higher scores among Arab compared with Chaldean respondents are consistent with prior studies that have indicated that Chaldeans may be more likely to assimilate into American society than their Arab, largely Muslim, counterparts [4]. Another reason for higher scores among Arab compared with Chaldean respondents is that some of the items only mentioned “Arab identity” and did not include language such as “Arab or Chaldean identity.” Chaldeans may have been less likely to endorse such items. Thus, future versions of the instrument, if intended for use among the Chaldean community, may benefit from more inclusive language.

We created a three-factor version of the measure that had good fit for both Arab American and Chaldean respondents. These common factors were useful in our study as we were interested in comparing results for the two groups and is recommended when researchers want to compare these two populations. It is possible, however, that unique versions of the measure could be developed for each group that may have different items or even subscales, particularly when direct comparison between groups is not required. Finally, although the factor analyses indicated three distinct subscales, researchers and practitioners can use the single-factor version when the three-factor approach may not fit with their objectives. The 20-item total scale score showed similar construct validity as the subscales.

In terms of health behaviors and health conditions, having higher MENA-IM scores was generally protective. Specifically, MENA-IM scores were associated with better mental health status as well as lower use of alcohol, marijuana, and prescription pain medication. Higher MENA-IM scores were also found among those reporting a history of heart disease. These findings of a protective effect between ethnic identity and substance use and health outcomes is consistent with some prior studies. For example, Jadalla et al. [27] found that attraction to Arabic culture, measured somewhat similarly as the MENA-IM, was positively associated with better stress management and nutritional practices. These positive health behaviors may partially explain the protective effect of ethnic identity on heart disease and mental health that we observed.

Of the three subscales, the one measuring a multicultural worldview had the most significant associations with better health (lower heart disease and better mental) and lower substance use. The items in this subscale appear to tap how much one views themselves not only as a member of their ethnic group but also as being part of the broader world community. This multicultural worldview may exert a positive protective effect by increasing connections with not only people from their own cultural but also people from other ethnic and cultural origins. Further work understanding the protective effect of a multicultural worldview is encouraged.

In our study, the protective effect of EI remained after adjustment for birth country, and there was no interaction between EI and birth country on any outcomes (data not shown). Abuelezam et al. [9], however, found that second-generation AAs had higher odds of binge drinking than first-generation immigrants, and third-generation AAs had higher odds of overweight/obesity than first-generation immigrants. EI may function independently from acculturation, or it could serve as a buffer for its deleterious effects [9]. Our data does not allow us to examine the interaction of EI and acculturation, although this warrants future research.

Another study [12] found that attraction to Arab culture was associated with higher cigarette smoking. This is inconsistent with our findings. We found that MENA-IM scores were unrelated to cigarette or hookah use. However, some of our scales were significantly associated with lower rates of marijuana and alcohol. Thus, we found that ethnic identity was protective for some substance use behaviors rather than a risk factor. These conflicting findings may indicate that the MENA-IM may be tapping unique dimensions of ethnic identity than prior measures. There are other differences between the current and prior studies that may also contribute to inconsistent findings including the fact that our study was conducted in Michigan, where an ethnic enclave exists, whereas most of the prior cited studies were conducted in California; the MENA communities differ in many ways between these states, and these differences may impact both ethnic identity and health behaviors [1, 2, 8, 20, 37].

Given the largely protective association we observed between MENA-IM scores and substance use and health outcomes, efforts to encourage younger and second-generation MENA populations to retain their culture and traditions may be warranted, particularly interventions that incorporate and enhance ethnic identity. This may include positive ethnic socialization delivered by parents and MENA cultural institutions and venues within mainstream institutions that provide socialization opportunities for individuals of similar ethnic backgrounds (e.g. youth groups, professional associations, student groups). Similar programmatic efforts have been conducted with other racial and ethnic group and would seem worthy of investigation and evaluation for the MENA community.

### Limitations and Future Directions

The survey used a community-based, convenience sample rather than a randomly drawn probability sample. Consequently, the sample recruited has several biases relative to the national and local Michigan MENA populations. For example, nationally, the median household income for MENA individuals is around $60,000, whereas in our sample, 69% reported income under $50,000. Our sample also appears to have lower educational attainment [2]. Nationally, 20% of Arab Americans have a post-graduate degree, compared with 11% in our sample [2]. This may be related to our sample’s relatively younger age. Thus, both the values we observed for the MENA-IM and their association with demographic and health variables may not generalize to the broader MENA population in Michigan or nationally. In addition, data were self-reported, which may impact the validity of key variables such as health habits, health history, and immigration status. We are unable to quantify a response rate, given the public spaces where participants were recruited. Thus, we cannot determine how respondents may have differed from those who declined to participate. Finally, we did not fully measure acculturation or generational status, only where respondents were born and what languages were spoken in the home. Understanding the association of the MENA-IM with acculturation merits future research.

Despite these limitations, the initial psychometric properties of the MENA-IM suggest that the measure may be useful in characterizing ethnic identity and elucidating psychosocial and physical health outcomes in MENA populations. Research examining how the measure performs with other MENA populations outside of Michigan and how it may relate to other health outcomes beyond those reported herein is encouraged.

## Data Availability

Data sharing via repositories supported, in accordance with University of Michigan’s institutional data sharing policies.
